# P-159. Epidemiological and Clinical Characteristics of Dengue Fever in Patients Admitted at a Universitary Hospital

**DOI:** 10.1093/ofid/ofae631.364

**Published:** 2025-01-29

**Authors:** Gustavo A Mendez, Jon Salmanton-Garcia, Carla Niveyro, Pedro A Villalba, Victoria Martin, Lucila Compañy, Maria C Tomasino, Carmen V Ferreyra, Flavia M Pelech, Lein H Kuo, Federico Irala, Celeste Davalos, Carlos G Veronesi, Agustina Varas, Oliver A Cornely, Karina A Salvatierra

**Affiliations:** Hospital Escuela de Agudos Dr. Ramon Madariaga, Posadas, Misiones, Argentina; University Hospital Cologne, Cologne, Germany, Cologne, Nordrhein-Westfalen, Germany; Argentine society of infectology, Posadas, Misiones, Argentina; Argentine society of infectology, Posadas, Misiones, Argentina; Argentine society of infectology, Posadas, Misiones, Argentina; Hospital Escuela de agudos Dr. Ramón Madariaga, Posadas, Misiones, Argentina; Infectious Diseases Department, Hospital Escuela de Agudos Dr. Ramón Madariaga, Posadas, Misiones, Argentina; Sanatorio IOT, Posadas, Misiones, Argentina; Sanatorio Boratti, Posadas, Misiones, Argentina; Hospital SAMIC Obera, Obera, Misiones, Argentina; Hospital SAMIC ElDorado, ElDorado, Misiones, Argentina; Hospital SAMIC ElDorado, ElDorado, Misiones, Argentina; Hospital SAMIC ElDorado, ElDorado, Misiones, Argentina; Sanatorio Boratti, Posadas, Misiones, Argentina; University of Cologne, Faculty of Medicine and University Hospital Cologne, Cologne, Nordrhein-Westfalen, Germany; Facultad de Ciencias Exactas, Químicas y Naturales, Posadas, Misiones, Argentina

## Abstract

**Background:**

Dengue is the fastest spreading mosquito-borne viral infection, which affects people living in the tropical and subtropical countries and cause severe clinical symptoms and possibly death. We aimed to determine the epidemiological and clinical characteristics of patients with dengue fever and to find mortality predictors.

**Methods:**

We conducted a retrospective observational study between November 2023 and April 2024 in five hospitals of Misiones, Argentina. Dengue virus infection was confirmed by a positive non-structural protein 1 test, IgM capture ELISA or RT-PCR. Clinical and laboratory patient details were collected by an electronic case report form.

**Results:**

A total of 289 patients were documented. Overall mean age was 50 years (15-88). Over half (56%) of the patients were female. Common comorbidities: hypertension (36%), age >60 years (31%) and diabetes mellitus (18%). Signs and symptoms at hospital admission: fever (82%), asthenia (47%) and myalgias (33%). Most frequent warning signs at admission were abdominal pain 29%, vomiting 23% and drowsiness 22%. Frequent hematological and biochemical abnormalities: Hto >30% (83%), leukopenia < 4000 cells/mm^3^ (48%), thrombocytopenia severe and very severe (25%), ALT or AST >200 iu (16%) and creatinine >2 mg/dl (16%). Complications at any time during patient hospitalization: renal failure 15%, diarrhea 9% and acute respiratory distress syndrome (ARDS) 9%. Patients who required admission to the ICU 15%. Dengue classification: without warning signs 27%, with warning signs 61% and severe dengue 12%. Mortality associated with dengue 7%. Admission to ICU (aHR: 5.81 95% [CI 2.29-14.7]), ARDS (aHR: 5.44 [95% CI 2.25-13.17]) and platelet transfusion (aHR: 10.46 [95% CI 3.92-27.89]) were independently associated with increased mortality.

**Conclusion:**

Our fatality rate was similar to the reported in other studies. Early recognition of warning signs and hematological monitoring is essential in order to detect patients at risk and offer them adequate early treatment.

**Disclosures:**

**Oliver A. Cornely, Prof. Dr.**, Abbott: Honoraria|Abbvie: Advisor/Consultant|Abbvie: Honoraria|AiCuris: Advisor/Consultant|Akademie fur Infektionmedizin: Honoraria|Al-Jazeera Pharmaceuticals/Hikma: Honoraria|amedes: Honoraria|AstraZeneca: Honoraria|Basilea: Advisor/Consultant|Biocon: Advisor/Consultant|BMBF: Grant/Research Support|Boston Strategic Partners: Advisor/Consultant|CIdara: Advisor/Consultant|CIdara: Expert Testimony|CIdara: Grant/Research Support|CIdara: Participation on a DRC or DSMB|CoRe Consulting: Stocks/Bonds (Private Company)|Deutscher Arzteverlag: Honoraria|DZIF: Grant/Research Support|EasyRadiology: Stocks/Bonds (Private Company)|EU-DG RTD: Grant/Research Support|F2G: Grant/Research Support|Gilead: Advisor/Consultant|Gilead: Grant/Research Support|Gilead: Honoraria|Grupo Biotoscana/United Medical/Knight: Honoraria|GSK: Advisor/Consultant|GSK: Honoraria|IQVIA: Advisor/Consultant|IQVIA: Participation on a DRC or DSMB|Janssen: Advisor/Consultant|Janssen: Participation on a DRC or DSMB|Matinas: Advisor/Consultant|MedPace: Advisor/Consultant|MedPace: Grant/Research Support|MedPace: Participation on a DRC or DSMB|Medscape/WebMD: Honoraria|MedUpdate: Honoraria|Menarini: Advisor/Consultant|Moderna: Honoraria|Molecular Partners: Advisor/Consultant|MSD: Grant/Research Support|MSD: Honoraria|MSG-ERC: Advisor/Consultant|Mundipharma: Advisor/Consultant|Mundipharma: Grant/Research Support|Mundipharma: Honoraria|Noscendo: Honoraria|Noxxon: Advisor/Consultant|Octapharma: Advisor/Consultant|Octapharma: Grant/Research Support|Pardes: Advisor/Consultant|Partner Therapeutics: Advisor/Consultant|Patent: US18/562644|Paul-Martini-Stiftung: Honoraria|Pfizer: Advisor/Consultant|Pfizer: Grant/Research Support|Pfizer: Honoraria|PSI: Advisor/Consultant|PSI: Participation on a DRC or DSMB|Pulmocide: Participation on a DRC or DSMB|Sandoz: Honoraria|Scynexis: Advisor/Consultant|Scynexis: Grant/Research Support|Seqirus: Advisor/Consultant|Seqirus: Honoraria|Seres: Advisor/Consultant|Shionogi: Advisor/Consultant|Shionogi: Honoraria|streamedup!: Honoraria|The Prime Meridian Group: Advisor/Consultant|Touch Independent: Honoraria|Vitis: Honoraria

